# Hybrid Reconstruction in Head and Neck Surgery: Integration of Virtual Planning, Navigation, and Robotic Microsurgery

**DOI:** 10.3390/jcm15082963

**Published:** 2026-04-14

**Authors:** Thomas J. Sorenson, Rebecca Lisk, Alexis B. Jacobson, Adam Jacobson, Jamie P. Levine

**Affiliations:** 1 Hansjorg Wyss Department of Plastic Surgery, NYU-Langone Health, New York, NY 10016, USA; thomas.sorenson@nyulangone.org (T.J.S.);; 2 Division of Head and Neck Surgery, NYU-Grossman School of Medicine, New York, NY 10016, USA; 3 Department of Otolaryngology-Head and Neck Surgery, NYU-Langone Health, New York, NY 10016, USA

**Keywords:** head and neck reconstruction, virtual surgical planning, CAD/CAM, robotic microsurgery, free flap reconstruction, surgical navigation

## Abstract

Reconstruction in head and neck surgery requires restoration of complex functions, including speech, swallowing, and breathing, while preserving as much facial form and patient identity as possible. Over the past decade, advances in preoperative digital planning, intraoperative technologies, and robotic platforms have reshaped reconstructive strategies, giving rise to the concept of hybrid reconstruction. Hybrid approaches integrate free tissue transfer with computer-aided design and manufacturing (CAD/CAM), virtual surgical planning, intraoperative navigation, and robot-assisted microsurgery to enhance precision, reproducibility, and functional outcomes. This narrative review examines the principles and applications of hybrid reconstruction in head and neck surgery with particular emphasis on osseous reconstruction of the mandible, maxilla, and midface. The roles of intraoperative navigation and robotic assistance as enabling tools are discussed, along with their potential benefits and current limitations. Functional and morphologic outcomes, patient-reported quality of life, and challenges related to cost, access, training, and evidence heterogeneity are critically reviewed. Hybrid reconstruction represents an advancement toward outcomes-driven, patient-centered care; however, thoughtful integration of emerging technologies and continued emphasis on rigorous outcome assessment are essential to guide responsible adoption in contemporary head and neck reconstructive surgery.

## 1. Introduction

Reconstruction of head and neck defects remains among the most technically demanding challenges in reconstructive surgery [1]. Surgeons must simultaneously restore essential functions, including speech, swallowing, and airway patency, while preserving facial morphology and the patient’s sense of identity [1,2]. Historically, reconstructive success was measured primarily by flap survival and defect coverage [3,4,5]. Contemporary reconstruction, however, has evolved toward a more nuanced objective: reliable restoration of function and form with reproducible outcomes and minimized morbidity [6,7]. Over the past decade, the reconstructive landscape has undergone substantial transformation. The increasing complexity of oncologic resections, combined with heightened expectations for functional and aesthetic outcomes, has driven adoption of advanced technologies, such as computer-aided design and manufacturing (CAD/CAM), virtual surgical planning, intraoperative navigation, robotic-assisted surgery, and emerging bioengineered solutions [8,9,10,11,12,13,14,15].

**Figure 1:**
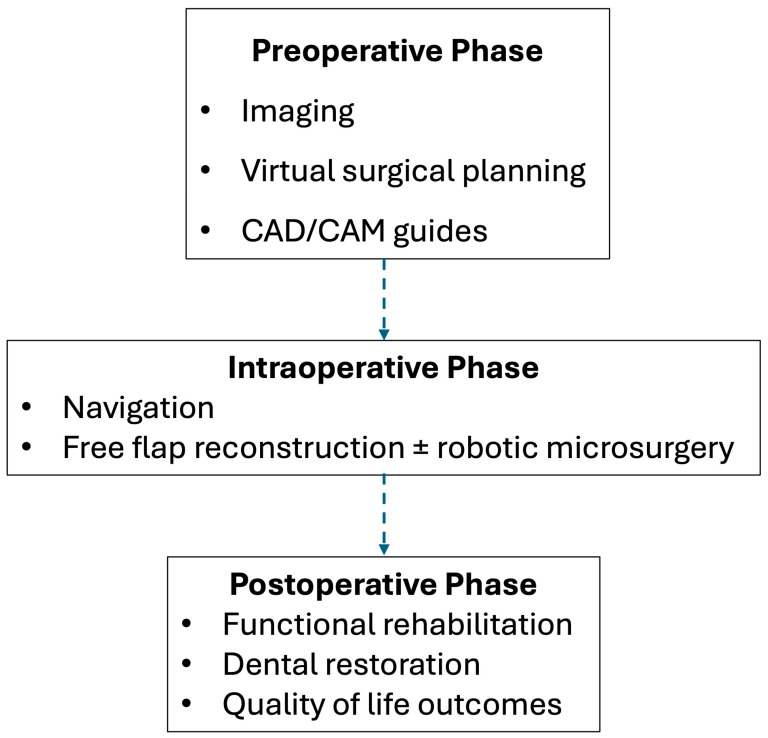
Framework of hybrid reconstruction.

Within this evolving framework, hybrid reconstruction has emerged as a unifying concept (Figure 1). Hybrid reconstruction refers to the integration of traditional free flap reconstruction with modern technological adjuncts. Hybrid approaches integrate established reconstruction techniques with digital planning and intraoperative technologies to enhance accuracy, efficiency, and functional outcomes [8,9,13,16]. This paradigm shift is particularly evident in osseous reconstruction of the mandible and midface where virtual planning and patient-specific implants have become increasingly common [17,18]. More recently, robot-assisted approaches have expanded the reconstructive armamentarium, enabling improved ergonomics and precision in select microsurgical applications [15,19,20,21].

To explore these emerging technologies, we performed a structured narrative review with PubMed/MEDLINE to identify relevant studies on emerging technologies in head and neck reconstruction, including three-dimensional surgical planning, intraoperative navigation, robotic-assisted microsurgery, and bioengineered tissues. This narrative review was conducted through a structured search of PubMed/MEDLINE, Embase, and Google Scholar for relevant literature published from 2000 to present. Search terms included combinations of “hybrid reconstruction,” “CAD/CAM,” “virtual surgical planning,” “intraoperative navigation,” “robotic microsurgery,” and “free tissue transfer.” Articles were selected based on relevance to reconstructive workflows integrating digital planning and intraoperative technologies, with emphasis on clinical studies, systematic reviews, and meta-analyses where available. Much of the current evidence base is limited by small sample sizes, heterogeneity in study design, and a predominance of retrospective and early feasibility studies, which restricts the ability to draw definitive conclusions regarding comparative effectiveness, and given the narrative nature of this review, formal systematic review methodology, including predefined inclusion/exclusion criteria and risk-of-bias assessment, was not performed. Both clinical and translational studies were included. Thus, this article presents a narrative review of hybrid reconstructive strategies in head and neck surgery with a focus on the integration of free tissue transfer, advanced planning technologies, intraoperative navigation, and robot-assisted microsurgery. Emphasis is placed on functional and morphologic outcomes, current challenges, and future directions in this rapidly evolving field.

## 2. Hybrid Reconstruction in Head and Neck Surgery

Hybrid reconstruction refers to the intentional integration of free tissue transfer with advanced digital and intraoperative technologies to optimize reconstructive accuracy, reproducibility, and patient-centered outcomes, including computer-aided design and manufacturing (CAD/CAM) as well as other novel technologies like virtual surgical planning, intraoperative navigation, and robot-assisted microsurgery [10]. Unlike traditional reconstruction, where only intraoperative decision-making and surgeon experience largely determine outcomes, hybrid reconstruction additionally relies on preoperative planning, technological augmentation, and multidisciplinary coordination to guide execution [10]. At its core, hybrid reconstruction acknowledges that modern head and neck defects often exceed the limitations of single-modality solutions [10]. Complex three-dimensional skeletal defects, composite soft-tissue losses, and functionally critical anatomical regions demand precise reconstruction that balances form, function, and durability [1,22,23,24]. Digital planning platforms enable surgeons to preoperatively simulate resections and reconstructions, anticipate challenges, and design patient-specific solutions [9,25]. Intraoperative navigation further translates these plans into the operative field with improved accuracy [13].

Robotic assistance represents a complementary extension of the hybrid paradigm. In head and neck reconstruction, robotic platforms offer potential advantages related to accessing to anatomically constrained spaces, enhancing visualization, and offering tremor reduction that may be advantageous in supermicrosurgical tasks [15,21,26,27]. While clinical adoption remains limited, early experiences suggest potential benefits in flap harvest and microsurgical anastomosis, particularly in ergonomically challenging scenarios [19,28]. In the following sections, we review these components, examine their application in osseous and soft-tissue reconstruction, and critically evaluate the evidence supporting their use in contemporary head and neck reconstructive surgery.

## 3. Medical Advances and Their Impact on Hybrid Reconstruction

Advances in systemic therapies and radiation delivery have significantly altered the oncologic landscape of head and neck cancer with important downstream implications for reconstructive surgery [29,30,31,32,33,34]. Improvements in chemotherapeutic regimens, targeted therapies, immunotherapy, and precision radiation techniques have enabled more effective tumor control while minimizing collateral damage to surrounding tissues [30,31,35,36,37]. As a result, surgical resections in selected patients have become more focused, facilitating reconstruction with reduced complexity and morbidity [38].

Contemporary chemotherapy and targeted systemic therapies have improved tumor response rates and local disease control, particularly in advanced-stage and previously unresectable disease [39,40,41]. Neoadjuvant treatment strategies may reduce tumor burden prior to surgery, allowing for more limited resections that preserve critical anatomic structures [42,43,44,45]. In some cases, improved oncologic responses even enables organ-preserving approaches or smaller composite defects, directly influencing reconstructive planning by decreasing the need for extensive chimeric or multilevel reconstruction [43].

Similarly, radiation therapy has undergone substantial refinement over the past decade. The widespread adoption of intensity-modulated radiation therapy (IMRT), image-guided radiation therapy, and adaptive radiation planning has improved conformality and reduced radiation exposure to adjacent normal tissues, which has several important implications [30,46,47,48,49]. Better preservation of recipient vessels and decreased surrounding soft tissue fibrosis expands reconstructive options and improves flap reliability, particularly in delayed or salvage settings [50,51]. Reduced radiation-associated tissue injury may also facilitate simpler soft-tissue reconstruction, decrease wound-healing complications, and lower rates of fistula formation [50,51,52]. In select cases, improved tissue quality even allows for reconstruction using smaller flaps or less extensive composite approaches [53,54].

Importantly, advances in oncologic therapy have also influenced the timing and sequencing of reconstruction. Enhanced systemic disease control and improved patient tolerance of multimodal therapy have enabled more predictable coordination between oncologic resection and reconstruction [55,56]. This predictability supports preoperative planning and integration of hybrid reconstructive strategies, allowing surgeons to tailor reconstruction to anticipated defect size and functional requirements with greater confidence [2,8,57].

Despite these benefits, modern oncologic therapies introduce new considerations for reconstruction. Immunotherapy and targeted agents may affect wound healing and inflammatory responses, while cumulative radiation dose remains a critical determinant of tissue quality [58,59,60,61]. As survival improves, reconstructive goals increasingly emphasize long-term functional durability and quality of life, necessitating careful consideration of how prior and planned therapies influence reconstructive choice [62,63]. Overall, advances in chemotherapy and radiation therapy have contributed to a shift toward smaller, more precise resections in appropriately selected patients, reducing reconstructive burden and enabling more tailored approaches [31,42,43,44,45,49,64]. These oncologic developments complement advances in hybrid reconstruction by creating an environment in which precision planning, focused reconstruction, and function-driven outcomes can be more consistently achieved [2,8,57,62,63].

## 4. Preoperative Digital Planning and Virtual Surgical Simulation

Preoperative digital planning has become a foundational component of hybrid reconstruction in head and neck surgery [8,25,65]. Computer-aided design and manufacturing (CAD/CAM)-assisted reconstruction refers to the use of virtual surgical planning and patient-specific guides or implants to enhance precision in otherwise conventional reconstructive workflows. This enable surgeons to transition from intraoperative improvisation toward predefined, patient-specific reconstructive strategies, particularly in complex osseous and composite defects [9,11,66].

Virtual planning platforms allow three-dimensional reconstruction of patient anatomy using preoperative imaging, facilitating precise assessment of defect geometry, occlusion, and spatial relationships [9,10,67,68,69,70]. In mandibular and midface reconstruction, this capability is especially valuable for defining osteotomy location, segment length, and orientation prior to surgery [68,69,70]. By simulating both resection and reconstruction, surgeons can anticipate reconstructive challenges and optimize flap design, fixation strategies, and implant selection before entering the operating room [8,10,25,65,68].

One of the principal advantages of digital planning is reproducibility. Patient-specific cutting guides and prebent or custom-fabricated fixation plates translate virtual plans into predictable intraoperative execution, reducing variability associated with freehand techniques [71,72,73,74]. Multiple high-level studies, including a systematic review of the literature, have demonstrated improved accuracy of bony reconstruction, restoration of mandibular contour, and occlusal alignment when CAD/CAM-assisted techniques are employed, particularly in complex or multi-segment reconstructions [71,72,73,75]. Beyond skeletal accuracy, digital planning contributes to operative efficiency. Predefined osteotomies and fixation strategies have been found to reduce ischemia time, shorten operative duration, and streamline multidisciplinary coordination between ablative and reconstructive teams [8,75,76,77,78,79]. These efficiencies are particularly relevant in high-risk patients or prolonged oncologic resections, where operative time and physiologic stress are critical considerations [80].

Despite these advantages, digital planning is not without limitations. Preoperative plans are inherently static and may not fully account for intraoperative findings such as unexpected tumor extent, tissue quality, or vascular variability [10,25,69,81]. Additionally, reliance on external vendors, production timelines, and increased costs may limit accessibility, particularly in resource-constrained settings [82,83,84]. Surgeon familiarity with planning software and interpretation of virtual models also introduces a learning curve that may affect early adoption [11,85].

Importantly, digital planning should be viewed as an enabling framework rather than a rigid protocol, particularly because oncologic resections may occasionally exceed anticipated margins. In these situations, surgeons must have additional plans in place as well as be able to fall back on their traditional surgical principles. The true value of CAD/CAM lies in its ability to enhance precision while preserving the surgeon’s capacity to adapt intraoperatively [25,86].

## 5. Hybrid Bone Reconstruction in Head and Neck Surgery

Osseous reconstruction represents one of the most technically demanding domains in head and neck surgery and has become the primary setting in which hybrid reconstructive principles have been most widely adopted [1,17,87]. Restoration of skeletal continuity, occlusion, facial projection, and load-bearing function often requires a level of precision that exceeds what can reliably be achieved with biologic tissue transfer alone [88,89]. As a result, hybrid bone reconstruction, integrating free osseous flaps with digital planning and technological adjuncts, has emerged as a contemporary standard for complex mandibular, maxillary, and midface defects [8,90,91].

### 5.1. Mandibular Reconstruction

Mandibular reconstruction exemplifies the evolution toward hybrid approaches. The fibula free flap remains the workhorse for mandibular defects due to its reliable vascular anatomy, length, and capacity for multi-segment osteotomies [92,93]. However, traditional freehand fibula reconstruction is limited by variability in osteotomy accuracy, plate contouring, and occlusal alignment [94,95,96,97,98,99]. CAD/CAM-assisted planning has addressed these limitations by enabling precise definition of defect geometry, osteotomy sequence, and fixation strategy prior to surgery [94,95].

Hybrid mandibular reconstruction typically combines virtual surgical planning, patient-specific cutting guides, and precontoured or custom fixation plates with free fibula transfer [73,100,101,102]. This integration improves alignment of bony segments, restoration of mandibular contour, and reproducibility of occlusion [18,68,86,103,104,105]. Small retrospective series have demonstrated improved accuracy and reduced intersegmental gaps when compared with conventional techniques, particularly in large or multi-segment defects [68,94,100,103]. Beyond geometric accuracy, hybrid approaches may enhance functional outcomes [105,106,107,108,109,110]. Improved occlusal alignment facilitates mastication and speech rehabilitation, while more anatomic mandibular contour contributes to facial symmetry and patient-reported satisfaction [105,106,107,108,109,110]. Importantly, hybrid planning also allows reconstruction to be designed with future dental rehabilitation in mind, aligning reconstructive goals with long-term functional restoration [88,105,106,107,108]. However, while improvements in geometric accuracy and surgical planning are consistently demonstrated, downstream benefits in functional outcomes and quality of life remain less well established and are supported by limited and heterogeneous data.

### 5.2. Maxillary and Midface Reconstruction

Hybrid reconstruction has also gained prominence in maxillary and midface defects, where three-dimensional complexity and proximity to critical structures pose substantial challenges [91,111,112,113,114]. Restoration of midface projection, orbital support, and palatal integrity is essential for speech, swallowing, ocular function, and facial aesthetics [112,115,116,117]. Virtual planning enables accurate reconstruction of skeletal buttresses and facilitates integration of free tissue transfer with patient-specific implants or fixation systems [113,118,119,120,121].

In maxillary reconstruction, hybrid strategies often combine free tissue transfer, such as fibula or scapular flaps, with CAD/CAM-designed plates or implants to reestablish midfacial architecture [91,114,122,123,124]. Digital planning allows precise positioning of bony segments relative to the cranial base and orbit, improving symmetry and reducing postoperative malocclusion or enophthalmos [119,121,123,125]. These benefits are particularly relevant in extensive defects or secondary reconstructions, where anatomic landmarks may be distorted or absent [125,126,127].

### 5.3. Functional Considerations in Hybrid Bone Reconstruction

A defining feature of hybrid bone reconstruction is its emphasis on functional restoration as a primary endpoint [128]. Skeletal accuracy alone is insufficient if reconstruction fails to support speech, swallowing, mastication, and airway stability [10]. Hybrid approaches facilitate preoperative consideration of functional outcomes by enabling surgeons to simulate occlusion, anticipate soft-tissue requirements, and coordinate multidisciplinary input from dental specialists, speech-language pathologists, and maxillofacial prosthodontists [78,129,130]. Additionally, hybrid reconstruction promotes consistency across cases, which is particularly valuable in high-volume centers and teaching institutions [11,85,131]. Standardized planning and execution may reduce variability in outcomes, improve efficiency, and enhance training by providing reproducible reconstructive frameworks [8,10,11,85,131].

### 5.4. Limitations and Current Challenges

Despite its advantages, hybrid bone reconstruction is not universally applicable. Increased costs, dependence on specialized infrastructure, and extended preoperative planning timelines may limit accessibility [25,82,83,84]. Furthermore, unanticipated intraoperative findings, such as altered resection margins or vascular anomalies, may necessitate deviation from preoperative plans [86,132]. In these scenarios, surgeon experience and adaptability remain paramount [86,132]. Hybrid bone reconstruction therefore represents an augmentation rather than a replacement of traditional reconstructive principles [10,25,85]. Its success depends on thoughtful integration of technology with sound surgical judgment, appropriate patient selection, and institutional support [25,85].

## 6. Intraoperative Technologies as Enabling Tools in Hybrid Reconstruction

While preoperative digital planning establishes the foundation for hybrid reconstruction, intraoperative technologies are essential for translating virtual plans into accurate and reproducible surgical execution [15]. In head and neck reconstruction, intraoperative navigation and robot-assisted techniques function as enabling tools that may be useful for bridging the gap between planning and performance, particularly in anatomically complex and spatially constrained reconstructions [13,15,133]. However, given the rapid evolution of these technologies, many proposed benefits remain theoretical or supported by limited early clinical data.

### 6.1. Intraoperative Navigation and Real-Time Guidance

Intraoperative navigation has emerged as an increasingly valuable adjunct in hybrid head and neck reconstruction, particularly for osseous reconstruction of the mandible, maxilla, and midface [13,127,134]. Navigation systems allow real-time spatial tracking of surgical instruments relative to preoperative imaging, facilitating accurate execution of planned osteotomies, implant positioning, and skeletal alignment [71,134,135,136,137].

When integrated with CAD/CAM planning, navigation enhances the fidelity of reconstruction by verifying that intraoperative steps adhere to the virtual plan [70,97,138]. This capability is particularly relevant in scenarios where anatomic landmarks are distorted or absent, such as revision surgery, secondary reconstruction, or extensive oncologic resections [127,138,139]. Navigation may also serve as a confirmatory tool, allowing surgeons to assess alignment and symmetry intraoperatively before definitive fixation [97]. Limited retrospective data have demonstrated improved accuracy of bone placement and reduced deviation from planned reconstructions when navigation is employed [13,70,97,140]. However, its routine use remains limited by increased operative setup time, equipment costs, and the need for institutional expertise [97,141].

### 6.2. Robot-Assisted Microsurgery

Robot-assisted surgery represents a more recent addition to the hybrid reconstructive paradigm, and robot-assisted approaches have been described in flap harvest, inset, and, microsurgical anastomosis [15,19,142,143,144]. In head and neck reconstruction in particular, robotic platforms have been reportedly utilized to improve access to anatomically constrained regions (e.g., transoral or deep head and neck defects), enhance visualization, and address ergonomic challenges inherent to microsurgical work [15,19,21,26]. Potential other indications for robotic-assisted microsurgery include supermicrosurgical procedures on particularly small or radiated/friable vessels and/or lymphatics in the head and neck region as well as cases where surgeon ergonomics may be significantly challenged by conventional approaches, like in the deep submandibular space. Potential advantages include motion scaling and tremor filtration for small and radiated vessels that require extra delicate handling, as well as improved surgeon ergonomics, which may be particularly beneficial in setting of surgeon height difference, during prolonged microsurgical anastomosis or in confined operative fields [15,19,145,146,147]. In reconstructive settings, robotic assistance may facilitate precise flap inset in deep oropharyngeal or skull base defects where conventional exposure is limited [28,148]. Barriers to widespread adoption include cost, system availability, extended operative times during the learning phase, and the need for specialized training [26,149,150,151].

Robot-assisted microsurgery remains in an early phase of clinical adoption, with limited but growing evidence supporting its feasibility [15,19,26,150]. Compared to conventional microsurgery, robotic-assisted techniques theoretically offer enhanced instrument stability and ergonomics; in our experience, we prefer to have an assistant under the microscope while the robotic surgeon performs the anastomosis. From the assistant perspective, the anastomosis is like assisting in conventional microsurgery; the main difference is to the robotic surgeon, who must learn the robotic system (Symani surgical system, Italy). However, at present, the published evidence supporting robotic microsurgery remains limited to early feasibility studies and small case series with no high-level comparative data demonstrating superiority over conventional techniques [19,150].

Importantly, robotic platforms must be viewed as adjunctive tools rather than replacements for microsurgical expertise [150]. Their role in hybrid reconstruction could be best understood as expanding the surgeon’s technical capabilities in selected scenarios, rather than redefining reconstructive principles [130,150].

### 6.3. Integration of Intraoperative Technologies Within Hybrid Reconstruction

The true value of intraoperative technologies lies in their integration within a broader hybrid reconstructive framework. Navigation and robotic assistance can be most effective when guided by thoughtful preoperative planning and applied selectively based on defect complexity, anatomic constraints, and institutional resources [152,153,154]. When used appropriately, these tools may be able to enhance reconstruction without undermining surgical adaptability [15,20,26,152]. As hybrid reconstruction continues to evolve, future studies will be necessary to define evidence-based indications for intraoperative technologies, assess their impact on long-term functional outcomes, and determine cost-effectiveness [26,152,155,156]. Until such data are available, adoption should remain judicious and outcomes-driven.

## 7. Functional and Morphologic Outcomes in Hybrid Head and Neck Reconstruction

As reconstructive paradigms have improved, contemporary head and neck reconstruction has begun shifting from a primary focus on defect coverage and flap survival toward a more comprehensive evaluation of functional and morphologic outcomes [16,157]. Speech intelligibility, swallowing efficiency, airway stability, facial symmetry, and patient-reported quality of life are now recognized as important reconstructive endpoints [158,159,160]. Hybrid reconstruction, by enhancing precision and reproducibility, has the potential to improve these outcomes across a range of complex defects [8,104,161].

### 7.1. Functional Outcomes

Functional restoration is central to successful head and neck reconstruction [88]. Accurate skeletal reconstruction plays a foundational role in speech articulation, mastication, and swallowing by reestablishing appropriate anatomic relationships between the mandible, maxilla, tongue, and pharynx [88,115,162,163]. Hybrid bone reconstruction facilitates precise restoration of occlusion and mandibular continuity, which in turn supports more effective oral intake and speech rehabilitation [70,105,108,134,164,165].

Several cohort studies, as well as systematic reviews, have suggested that CAD/CAM-assisted reconstruction may improve early functional outcomes by reducing malocclusion, segmental misalignment, and instability [8,11,12,90,166,167]. Improved accuracy may also facilitate earlier initiation of oral feeding and speech therapy, particularly in complex mandibular and midface reconstructions [12,164,166]. While long-term functional data remain limited, early results support the role of hybrid approaches in enhancing functional predictability [65].

Airway management is another critical consideration, particularly in extensive midface or mandibular reconstructions [168,169,170]. Accurate skeletal positioning and soft-tissue support may reduce the risk of airway compromise and improve long-term airway stability [171,172,173,174]. In select cases, hybrid planning enables anticipation of airway challenges and coordination of reconstructive strategies that minimize postoperative respiratory morbidity [125,175,176].

### 7.2. Morphologic and Aesthetic Outcomes

Preservation and restoration of facial morphology are essential to patient identity and psychosocial well-being [7,177,178]. Hybrid reconstruction enables more accurate restoration of facial contours, projection, and symmetry by replicating pre-morbid anatomy through virtual planning [9,11,125,179]. This precision is particularly valuable in the midface and mandible, where small deviations in skeletal alignment can result in significant aesthetic asymmetry [104,176].

Patient-specific implants and precontoured fixation systems contribute to consistent facial contouring, reducing reliance on intraoperative estimation [180,181,182,183]. Improved morphologic outcomes may translate into higher patient satisfaction and reduced need for secondary revision procedures, although high-quality comparative data remain limited [166,181]. Importantly, hybrid reconstruction also supports planning for secondary aesthetic refinements and dental rehabilitation [106,107,184,185,186]. By restoring skeletal architecture with greater accuracy, subsequent interventions, such as dental implants or soft-tissue contouring, can be performed more predictably, contributing to long-term aesthetic and functional success [106,107].

### 7.3. Patient-Reported Outcomes and Quality of Life

Patient-reported outcome measures (PROMs) are increasingly recognized as valuable in evaluating reconstructive success [187,188]. While data specific to hybrid reconstruction are limited, limited cohort data suggests that improvements in functional and morphologic accuracy correlate with enhanced quality of life and patient satisfaction [8,106,109,189,190]. Hybrid approaches may reduce variability in outcomes, which is particularly relevant for patients undergoing extensive or staged reconstruction [8,10,11,85,131].

Although patient-reported outcomes are increasingly recognized as important in reconstructive evaluation, data specific to emerging technologies in head and neck reconstruction remain sparse and heterogeneous with inconsistent use of validated PROMs and limited long-term follow-up [11,62,109,191]. Most available studies do not incorporate validated instruments or are underpowered to detect meaningful differences; future studies incorporating standardized outcome measures will be valuable to fully defining the patient-centered benefits of hybrid reconstruction.

## 8. Challenges, Limitations, and Controversies in Hybrid Head and Neck Reconstruction

Despite the growing adoption of hybrid reconstructive strategies, significant challenges and unresolved controversies remain. While advanced technologies offer clear advantages, their integration into routine clinical practice raises important questions regarding cost, accessibility, training, and evidence-based utilization.

### 8.1. Cost, Resource Utilization, and Access

One of the primary barriers to widespread adoption of hybrid reconstruction is increased cost. Virtual surgical planning, patient-specific implants, intraoperative navigation, and robotic platforms require substantial financial investment and institutional infrastructure [82,83,84]. These costs may be difficult to justify in settings with limited resources or in healthcare systems under increasing pressure to demonstrate value-based care [82,83,84]. Furthermore, access to hybrid technologies is uneven across institutions and geographic regions. High-volume tertiary centers are more likely to possess the necessary expertise and equipment, potentially exacerbating disparities in reconstructive care [192,193]. The absence of standardized cost-effectiveness data further complicates decision-making, as improved accuracy does not always translate directly into measurable economic or clinical benefit [8,65,180,194].

### 8.2. Training, Learning Curves, and Workflow Integration

Hybrid reconstruction introduces additional complexity into surgical workflows and training paradigms. Surgeons must acquire familiarity with digital planning platforms, navigation systems, and robotic interfaces, often requiring collaboration with engineers and industry partners [9,10,179,195]. These learning curves may initially increase operative times and complicate team coordination [10,25]. In training environments, the balance between standardization and surgical education presents a particular challenge. While hybrid planning can enhance reproducibility, excessive reliance on preoperative plans may limit opportunities for trainees to develop intraoperative decision-making skills [25,85,196]. Ensuring that technology augments rather than replaces foundational reconstructive principles is critical for sustainable adoption.

### 8.3. Evidence Gaps and Outcome Heterogeneity

Despite increasing adoption of hybrid reconstructive strategies, the current evidence base remains limited by methodological heterogeneity and a predominance of lower-level studies. Much of the available literature consists of retrospective analyses, single-institution experiences, and early feasibility studies with relatively small sample sizes and inconsistent reporting standards [66,83,161,191,197]. These limitations constrain the ability to draw definitive conclusions regarding comparative effectiveness and generalizability across diverse clinical settings.

A major challenge lies in the absence of standardized outcome measures. Studies evaluating hybrid reconstruction frequently employ heterogeneous endpoints, including geometric accuracy, operative efficiency, complication rates, and functional outcomes, which complicates cross-study comparisons and meta-analyses [150]. Furthermore, inconsistencies in the use of validated patient-reported outcome measures (PROMs) limit the assessment of patient-centered benefits such as speech, swallowing, aesthetics, and quality of life [62]. The lack of consensus regarding clinically meaningful endpoints underscores the need for standardized reporting frameworks and core outcome sets tailored to head and neck reconstruction.

High-quality comparative data are also scarce. While computer-aided design and manufacturing, intraoperative navigation, and robotic-assisted techniques have demonstrated improvements in precision and workflow efficiency, robust prospective trials comparing hybrid approaches with conventional reconstruction remain limited [166]. Long-term functional outcomes, cost-effectiveness, and oncologic safety have not been uniformly evaluated [166]. Randomized controlled trials and multicenter prospective studies are necessary to clarify the true clinical value and durability of these technologies.

The evidence supporting robot-assisted microsurgery is especially nascent. Current reports primarily consist of small case series and feasibility studies, with insufficient data demonstrating superiority over traditional microsurgical techniques in terms of clinical outcomes or cost-effectiveness [21,28,198,199]. Without high-quality comparative trials, it remains unclear which patient populations derive meaningful benefit from robotic assistance in reconstruction.

Additional gaps exist in understanding the economic and systemic implications of hybrid reconstruction. Comprehensive cost–utility analyses and value-based assessments are limited, particularly in diverse healthcare systems and resource-constrained environments [82,83,84]. These uncertainties raise important considerations regarding equitable access, scalability, and responsible integration into clinical practice.

Finally, the rapid pace of technological innovation presents inherent challenges to evidence generation. Advances in artificial intelligence, bioengineered constructs, and next-generation robotic systems may outpace the maturation of long-term clinical data, resulting in an evolving evidence landscape [200,201,202]. Continuous reassessment through prospective registries, standardized data reporting, and multidisciplinary collaboration will be essential to ensure that technological adoption remains evidence-based and patient-centered.

### 8.4. Risk of Technology-Driven Overuse

A central controversy in hybrid reconstruction is the potential for technology-driven overuse. The availability of advanced tools may encourage their application in cases where conventional techniques would suffice, without clear evidence of added benefit [176,203]. This risk underscores the importance of outcomes-driven adoption and careful patient selection [203]. Hybrid reconstruction should not be viewed as a universal solution, but rather as a selective strategy for complex defects that justify technological augmentation [10]. Maintaining this distinction is essential to avoid unnecessary escalation of cost and complexity [10].

## 9. Future Directions in Hybrid Head and Neck Reconstruction

Hybrid reconstruction in head and neck surgery continues to evolve as technological innovation, multidisciplinary collaboration, and outcome-driven practice converge. Future advances are likely to focus on improving personalization, and efficiency, while addressing current limitations in access and evidence generation [204,205,206]. Artificial intelligence–assisted planning represents a promising extension of digital reconstruction [201,202,207]. Machine learning algorithms may enhance preoperative planning by optimizing osteotomy design, predicting functional outcomes, and streamlining workflow integration [200,201,202,207,208]. Automation of routine planning steps could reduce reliance on external vendors and shorten preoperative timelines, increasing accessibility [208,209]. Advances in bioengineered tissues and scaffold technologies may further expand the hybrid paradigm [210,211,212,213]. The integration of bioresorbable or bioactive constructs with free tissue transfer has the potential to improve tissue regeneration, reduce donor-site morbidity, and enhance long-term reconstructive durability [16,214,215]. As these technologies mature, hybrid reconstruction may increasingly combine biologic and engineered solutions in a patient-specific manner.

Robot-assisted microsurgery is also likely to evolve as platforms become more refined and specialized for reconstructive applications. Improvements in haptic feedback, instrument dexterity, and microsurgical precision may expand the role of robotics beyond selected cases [21,27,150,216]. Future comparative research should prioritize defining evidence-based indications, evaluating functional outcomes, and assessing cost-effectiveness to guide responsible adoption. Finally, standardized reporting of functional and patient-reported outcomes will be essential to advancing the field [217]. Consensus-driven outcome measures will enable meaningful comparison across reconstructive strategies and facilitate evidence-based integration of emerging technologies [217].

## 10. Conclusions

Hybrid reconstruction represents a paradigm shift in head and neck reconstructive surgery, reflecting a broader transition from survival-focused care toward restoration of function, form, and patient identity. By integrating free tissue transfer with digital planning, intraoperative navigation, and robot-assisted techniques, hybrid approaches enhance precision, reproducibility, and functional predictability in complex reconstructions. Importantly, technology should serve as an adjunct to, rather than a replacement for, sound surgical judgment. Thoughtful, outcomes-driven application of hybrid strategies is essential to maximize benefit while minimizing unnecessary complexity. As the field continues to evolve, continued emphasis on multidisciplinary collaboration, rigorous outcome assessment, and patient-centered care will define the future of head and neck reconstruction.

## Data Availability

No new data were created or analyzed in this study.
